# Assessment of Environmental Impacts from Different Perspectives—Case Study of Egg Value Chain System in Serbia

**DOI:** 10.3390/foods11121697

**Published:** 2022-06-09

**Authors:** Marija Mitrovic, Igor Tomasevic, Ilija Djekic

**Affiliations:** Faculty of Agriculture, University of Belgrade, Nemanjina 6, 11080 Belgrade, Serbia; tbigor@agrif.bg.ac.rs (I.T.); idjekic@agrif.bg.ac.rs (I.D.)

**Keywords:** life-cycle assessment, environmental impacts, egg supply chain, sustainable consumption, environmental indicators

## Abstract

The environmental performance of various aspects of animal origin food supply chains has been the focus of research in recent years, and has provided useful information. However, there were no studies that covered the entire egg supply chain from different perspectives. The aim of this study was to analyze the majority of environmental impacts in the table egg supply chain comprising of three subsystems: farms, retail outlets and households, with quantification of each individual subsystem and the entire supply chain. All data were gathered from 30 farms, 50 retail stores and 300 households in Serbia. In parallel, the perception and ranking of environmental impacts along the supply chain were also evaluated. Finally, the quality function deployment for the environment was used to determine the degree of correlation between the set of environmental requirements and the identified environmental impacts. Results revealed that the greatest environmental impacts come from the production of feed for laying hens and the use of natural resources, and they contribute the most to the pollution of each individual environmental indicator. Additionally, the results show the differences in the environmental impacts of each individual subsystem and identify opportunities to mitigate them through the optimization of animal feed, energy consumption and household food waste management. The overall perspective of the egg supply chain points to climate change effects as the most important. The differences in the perceptions of environmental impacts along the entire egg supply chain suggest the need for promotion of mitigation strategies to all stakeholders that would encourage them to achieve sustainable development goals.

## 1. Introduction

Egg production has increased significantly in recent decades worldwide and is one of the fastest growing industries in the food sector [1]. Along with the rapid growth of this industry, general issues that need to be taken into account when planning the modern production of table eggs continue to emerge, such as: accelerated human population growth, increasing consumer demand for safe and quality food, limited natural resources and decreased environmental impact [2].

As the effects of faster industrial development on climate change intensify, great efforts are being made around the world to implement and achieve the 17 global Sustainability Development Goals (SDGs) of the United Nations [3]. The conclusions of the COP26 conference in Glasgow, at which the leaders of the world’s largest countries presented plans to switch to renewable energy sources and reach zero carbon emissions by 2050, was to support developing countries and increase the involvement of scientific communities in resolving the global environmental crisis [4]. Research by Van Berkum et al. emphasizes the importance of integrating all activities and actors within the food value chain through a holistic approach, as a key analytical framework to support strategic management decision-making associated with sustainable development of food systems [5]. To implement the sustainability strategy in the food industry, in addition to assessing the socio-economic situation, it is necessary to analyze the current state of environmental performance in all parts of the food system (not only in agricultural production), through constant monitoring, finding opportunities to reduce impact and affirming all stakeholders to recognize their roles in achieving SDG goals [6,7,8].

The table egg industry has undergone major reorganizations in recent years in order to adapt to the concept of sustainable development; thus, the industry is increasingly recognizing its role and responsibility in this area. One of the common applications is the Life Cycle Assessment (LCA) method, which, along with other tools and approaches, is used to assess the sustainability of table egg production. Interest in the application of the LCA method in the production of table eggs has lasted for more than a decade and is becoming more intense, but mainly in highly developed countries (briefly overviewed in Table 1). The research conducted assessed the most important environmental impacts by observing different phases of production, with the authors focusing mainly on the type of production system of laying hens [9]. The formulation of feed for laying hens has stood out as a very expensive input element and the most important category of impact on the environment in the poultry sector. The diet of laying hens is adjusted based on the availability of ingredients, price and transport costs so as to obtain the most optimal nutritional efficiency of the entire production. On the other hand, for the production of feed, it is necessary to cultivate the land for each of the crops that are part of it, use chemicals for soil treatment, fossil fuels for mechanization, water for irrigation, etc., which further increases the environmental impact in this part of the chain [10,11,12]. Although the dimensions of the environment were recognized as very important factors for research into animal origin food chains, there was no research that included all actors in the table egg chain: producers, retailers and household consumers. Additionally, there were no studies that examined actors’ perceptions of the environment in relation to the role that actors have in the supply chain.

In order to meet the latest challenges, the egg industry should develop in a sustainable way in order to increase production and reduce costs and environmental impacts, while respecting consumer demands for high quality eggs that will meet their expectations [2]. In addition, the environmental dimension of sustainability is becoming increasingly important when making decisions about buying eggs [13]. Therefore, the long-term future of table egg production cannot be estimated only by analyzing the impact of industrial systems without taking into account the analysis of consumption patterns [14]. One of the models that combines the two mentioned concepts is the Quality Function Deployment for Environment (QFDE) method, developed by Masui et al. [15], which refers to product planning while respecting environmental requirements [16]. Regardless of the fact that environmental sustainability strategies in the production of table eggs will have different imperatives in developing countries and highly developed countries, they certainly all face similar problems on the way to achieving targets associated with the UN SDGs [6]. For these reasons, the modern table egg industry needs to pay more attention to understanding and managing all processes and activities along the entire supply chain, from farm to household, to ensure sustainable development and respond to market demands.

Regardless of the importance of the environmental performance of the modern table egg industry, there have been no studies involving the entire supply chain in Serbia or worldwide from different perspectives. Therefore, to the best of our knowledge, this research provides better insight and support in assessing the complete product life cycle. The study includes environmental indicators and critical performance points that can be applied to other locations in different contexts along with other tools.

**Table 1:** An overview of Life Cycle Assessment (LCA) research in the egg chain in the last ten years.

No.	Authors	Country	Research Focus	System Boundaries			GWP [kg CO_2_eq/FU]	CED [MJ_e_/FU]	ODS [mg R11_e_/FU]	HTP [kg 1.4 DB_e_/FU]	AP [g SO2_e_/FU]	EP [g PO4_e_/FU]
				1	2	3						
1	Wiedemann and McGahan [17]	Australia	The impact of egg production in cages and from free-range on greenhouse gas emissions, and water and energy consumption.	√	-	-	Cage 1.3 ± 0.2 Free range 1.6 ± 0.2	0.7 ± 0.9 13.1 ± 1.1	-	-	-	-
2	Leinonen et al. [18]	United Kingdom	The impact of four different production systems (cage, barn, free range and organic) on global warming potential, acidification, eutrophication and energy consumption.	√	-	-	Cage 2.92 ± 0.21 Barn 3.45 ± 0.26 Organic 3.42 ± 0.34 Free range 3.38 ± 0.27	16.88 ± 1.1 22.20 ± 1.2 26.41 ± 1.6 18.78 ± 1.1	-	-	53.14 ± 5.2 59.43 ± 5.9 91.63 ± 8.6 64.13 ± 6.9	18.47 ± 1.5 20.32 ± 1.7 37.61 ± 4.2 22.03 ± 2.0
3	Pelletier et al. [19]	United States	The impact of intensive egg production and processing on greenhouse gas emissions.	√	-	-	5.0 (4.23–5.99)	-	-	-	-	-
4	Nielsen et al. [20]	Denmark	The impact of organic egg production on greenhouse gas emissions.	√	-	-	1.52 (1.34–1.82)	-	-	-	-	-
5	Taylor et al. [21]	United Kingdom	The impact of free-range egg production in small commercial units on mixed farms on greenhouse gas emissions.	√	-	-	1.6–1.8	-	-	-	-	-
6	Pelletier et al. [22]	United States	Comparison of the ecological impact of egg production in the period 1960 to 2010.	√	-	-	1960 year 7.23 2010 year 2.08	18 12	-	-	200 70	70 20
7	Ghasempour and Ahmadi, [23]	Iran	Ecological impact of laying hen diet in intensive egg production.	√	-	-	4.07	30.09	0.12	8.80	43.89	5.42
8	Pelletier, [9]	Canada	Comparison of the impact of five different egg production systems on the environment.	√	-	-	Cage 2.44 Enriched cage 2.30 Free run 2.40 Free range 2.39 Organic 1.36	11.24 12.06 11.81 11.47 7.95	-	-	78.4 82.5 80.6 69.8 46.6	24.4 26.8 25.6 23.8 14.8
9	Vetter et al. [24]	United States	Exploring the potential for reducing emissions in organic egg production using a GHG calculator.	√	-	-	1–5 (total 0.7–1.8)					
10	Abín et al. [1]	Spain	The impact of intensive egg production on the environment (carbon footprint).	√	-	-	3.4	-	-	-	-	-
11	Estrada-González et al. [25]	Mexico	Implementation of an eco-efficient scheme to reduce the environmental impact of egg production farms.	√	-	-	Egg posture phase 4.4 Total 5.6	-	-	0.14 0.16	-	-

GWP—global warming potential; CED—cumulative energy demand; ODS—ozone depletion substances; HTP—human toxicity potential; AP—acidification potential; EP—eutrophication potential; FU—functional units 1 kg of eggs. System boundaries: 1—farm, 2—retail, 3—household use; √—subsystem covered by the LCA study; subsystem was not covered by the LCA study.

Given the above, the aims of this study were to: (1) measure the following environmental indicators of the egg supply chain: energy and water consumption, global warming potential, ozone depletion potential, human toxicity potential, acidification and eutrophication potential based on established functional units, and; (2) examine perceptions of the environmental impacts throughout the chain, starting with producers, through retailers and end consumers of eggs (and vice versa) to analyze current practice by developing a two-way model.

## 2. Materials and Methods

### 2.1. Life Cycle Assessment (LCA)

The LCA of the entire table egg supply chain was performed on the basis of the methodology from the international reference standard ISO 14,040 in several phases (Table 1) [26].

#### 2.1.1. Determining the Subject and Scope of the LCA Study

**Figure 1:**
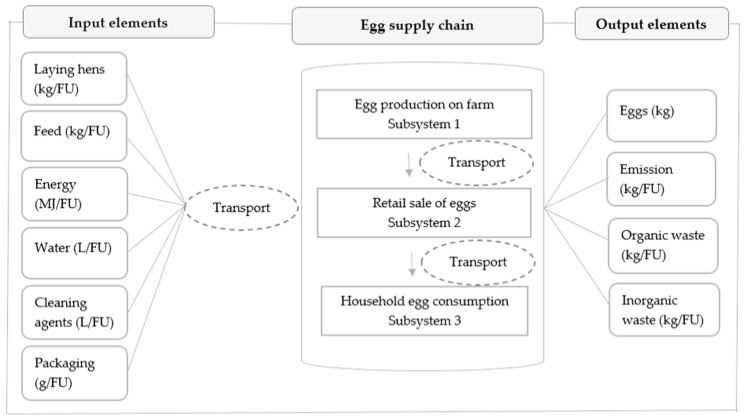
System boundaries of the egg production life cycle and material flow analysis; FU—Functional unit (1 kg of eggs).

The table egg supply chain was studied through three subsystems: production on farm, retail and household consumption (Figure 1). For each of the subsystems, environmental indicators based on a defined functional unit (FU) of 1 kg of eggs were identified. The burden on the environment was considered through the consumption of natural resources (water and energy), transport activities, waste management and greenhouse gas (GHG) emissions. The environmental impact of each individual subsystem and finally the overall impact of the whole chain were assessed to identify those critical points that could provide an opportunity for improvement [27]. Producers and retail outlets (large, medium, small) were selected to cover at least 50% of the market share in the production and trade of chicken eggs in Serbia, respectively [28].

The working hypotheses on which the study is based are: (1) actors (producers, retailers and consumers) differently perceive the environmental impacts of the egg supply chain; (2) the environmental impacts of actors, i.e., producers, retailers and consumers, could differ; (3) it is possible to reduce the environmental impact of the egg supply chain if the whole chain is considered as one system from the subsystem (production, retail, consumer) level.

#### 2.1.2. Inventory Analysis

Field data collection was conducted during 2021 through targeted LCA questionnaires for all actors in the egg supply chain. Before answering the questionnaire, all actors were informed about the purpose of the research, confidentiality of data and anonymity. Before proceeding, all actors gave their verbal consent. Data were collected in direct conversations with producers, quality/food safety managers of retail chains and household members.

For the purpose of collecting data, three questionnaires have been developed for each actor in the supply chain, based on similar research on different food supply chains [27,29,30]. The LCA questionnaire for producers on the farm referred to characteristics of production (capacity, type, selection of hybrids, breeding and transport of laying hens), the provision and type of feed (production, mode and dynamics of transport, consumption), use of natural resources (type and dynamics), manner of maintaining hygiene in the facility (type and quantity of water and cleaning agents), weight and share of each quality class of eggs produced, method of packaging and distribution to retail (type and quantity of material, transport), non-compliance that resulted in discarded eggs during production, dead hens, generated waste (organic and inorganic) and manure handling. The questionnaire for retailers consisted of questions related to annual turnover, sale and write-off of eggs, transport, consumption of natural resources during handling, storage and sale of eggs, ways of maintaining hygiene and refrigeration, type and amount of waste generated. Consumers in the household answered questions regarding weekly purchases (number of visits to retail outlet, method of travel to retail and distance of the travel to retail), weekly consumption of eggs (preparation time, method of preparation, amounts of water, oil and electricity used), egg storage conditions (number of days, models/type of refrigerator, refill of refrigerant) and manner of handling the generated waste.

Subsystem 1—egg production on farm: included all activities of rearing or procurement of laying hens until the 18th week, the phase of exploitation (egg transfer) from the 72nd to the 76th week and distribution of eggs to retail chains. The data collected were: type of production system, disinfection and cleaning, selection of laying hens, feeding and watering, packing eggs, transport, waste management. The research included 30 producers (large, medium and one part of small farms) that produce 62.6% of the total annual egg production in Serbia [28].

Subsystem 2—egg retail: included the retail practices covering the conditions of egg storage, maintenance of hygiene in the facility, transport from the main distribution retail center to retail stores and waste management. The research included 50 retail stores that account for 70.2% of the total annual egg sales in Serbia [28]. Large retail chains (which own 100 to 400 facilities) answered questions as one team by having their management representative fill out the questionnaire.

Subsystem 3—household consumption: data included the transportation mode after purchase (from retail to household), egg storage conditions, egg preparation for consumption and handling of generated waste (packaging and eggshell). A total of 300 households from urban parts of Serbia participated in the research, 50% from the capital Belgrade (where the food market is the largest and most developed), and the other 50% from the remaining regions of Serbia.

Assumptions: for households, the energy consumption of home refrigerators was assumed in the range of 100 to 150 kWh depending on the type, age and size. Electric furnaces ranged from 1500 to 2500 kWh depending on the type, age and size.

The inventory used for the three subsystems in the egg supply chain associated with 1 kg of eggs is presented in Table 2. It summarizes the data sources considered in this study based on the CCaLC and Ecoinvent© database [31] as well as data prescribed in Serbian regulations [32].

#### 2.1.3. Calculation and Analysis of Environmental Impact

The data obtained from the inventory analysis were calculated in relation to the total annual egg production for the producer subsystem, then to the total annual turnover of eggs for the retail subsystem and the total annual consumption of eggs in the consumer households. The calculated environmental impact indicators in the egg chain included global warming potential (GWP), cumulative energy consumption (CED), ozone depleting substances (ODS), human toxicity potential (HTP), acidification potential (AP) and eutrophication potential (EP). The calculation of the obtained data from all three subsystems was performed using the CCaLC and Ecoinvent© databases [27]. Statistical processing of the obtained data was performed in SPSS Statistics 20.0 (IBM SPSS Statistics for Windows, Version 20.0, IBM corporation, Armonk, NY, USA) using ANOVA one-factor analysis of variance. Differences between subsystems were found at the level of statistical significance of 0.05.

### 2.2. Customer-Supplier Interaction

Environmental aspects of the three main actors in the egg supply chain were identified on the basis of previous research related to the production of table eggs. Actors ranked in order of importance 12 identified environmental aspects related to their role in the egg supply chain and another 12 environmental aspects related to their direct suppliers, in order to compare the perceptions of the three subsystems towards the environmental impacts. The ranking by importance was performed from 1 (the least impact on the environment) to 12 (the greatest impact on the environment). After completing the field research, the average rankings for each environmental aspect in the direction from farm to household (bottom-up) were calculated. The analysis included farm–retail and retail–household interactions. The Mann–Whitney (U) test was used to determine ranking differences at a statistical significance level of 0.05, as in the work of Mitrovic et al. [33].

### 2.3. Production Planning with Respect to Environmental Requirements

Based on the work of Djekic et al. [5], four main UN SDGs associated with the food supply chain have been defined as key goals in evaluating environmental impacts in the table egg supply chain. Environmental requirements in the production of chicken eggs and the requirements’ relationships with environmental impacts is presented through the QFDE matrix in the form of a house of quality (HoQ) [34].

The first step in forming a HoQ was to identify and select environmental requirements in response to the question of what goals chicken egg production should meet (WHAT), while ranking the importance of the defined UN SDGs. Next, significant environmental impacts of all actors in the egg chain were identified through the three subsystems (HOW). The established correlation of importance between environmental requirements and environmental impact is shown by numbers through the relationship matrix [35].

The modified HoQ model shown describes the extent to which the identified environmental impacts (HOW) supported the achievement of set goals (WHAT) along the entire production chain. Data for ranking the importance of environmental requirements (*Q_i_*) were obtained from the field research (HOW) and represent a further basis for determining the importance of weight (*W_i_*) of identified environmental impacts ‘*i*’, determined by each of the actors in the egg supply chain. Relative weight calculations are shown by Equation (1):

(1) $$RW_{i}=\frac{W_{i}}{\sum_{i}^{n}W_{i}}\times 100\;\left(\%\right)$$

Relevant environmental impacts (*H_j_*) were identified for all actors in the egg supply chain (HOW). The degree of correlation between the set environmental requirements and the identified environmental impacts (*RS_ij_*) was determined as follows: ‘0′—no correlation; ‘1′—weak; ‘3′—medium; and ‘9′—strong correlation [36,37].

An expert team of scientists in the field of environmental protection performed the ranking of relationships in the formed HoQ matrix. Consensus on the level of correlation was reached with the help of the Delphi method, after a 60 min analysis was conducted [38].

The calculation of the absolute weight of each identified environmental impact (‘*n*’—number of activities affecting the environment through each subsystem) is shown by Equation (2):

(2) $$AW_{j}=\sum_{i=1}^{n}W_{i}\times RS_{ij}$$

*W_i_* represents the importance of the weight (WHAT) of, ‘*i*’, environmental requirements (*n*—number of identified requirements); *RS_ij_* shows the connection between the results of WHAT and HOW. The relative absolute importance of weight (RAW) was calculated from absolute weight and presented through one HoQ model for all actors in the egg supply chain [35].

## 3. Results and Discussion

### 3.1. Assessment of the Impact of the Egg Supply Chain on the Environment

Given that egg production on the farm is a technologically demanding and complex process, its environmental impact in terms of GWP, CED, ODS, HTP, AP and EP was the most significant observed throughout the chain (Table 3). The GWP of (2.63 kg CO_2_eq/kg) eggs was close to that obtained by Canadian authors (2.4 kg CO_2_eq/kg eggs) [9]. Slightly lower values were found in studies conducted in the UK (2.92–3.45 kg CO_2_eq/kg eggs) [18], Spain (3.4 kg CO_2_eq/kg eggs) [1] and Iran (4.07 kg CO_2_eq/kg eggs) [23]. The CED was in the range of (17.42 to 34.98 MJ_e_/kg egg) which is in line with results from the UK (16.88 to 26.41 MJ_e_/kg egg) [18], but significantly higher than the results obtained in Canada (7.95 to 12.06 MJ_e_/kg egg) [9]. The dominant form of energy used on the farm in Serbia is electricity for the maintenance of production systems for laying hens. Factors that affect the rate of electricity consumption are numerous and include the height of the temperature in the facility, the duration of daylight in hours and the intensity of lighting, capacity and facility layout, as well as the level of automation of production. The energy used for transport was most often fossil fuel, diesel fuel and liquefied petroleum gas. The values for ODS and HTP were (0.15 mg R11_e_/kg egg) and (1.04 kg 1.4 DB_e_/kg egg), respectively, which were close to the results reported in Iran [23]. AP on farms in Serbia ranged from (11.13 to 19.85 g SO2_e_/kg egg), so were significantly lower than AP values of studies from the UK, Iran and Canada [9,18,23]. EP ranged from (18.97 to 34.37 g PO4_e_/kg egg), and corresponded to the interval of results from the UK and Canada [9,18]. The main contribution of AP and EP is the use of nitrogen fertilizers in the production of feed for laying hens and the management of manure. The environmental impact on the farm was the greatest, but our results indicate that the impact of other parts of the supply chain should not be neglected. Recent research has shown that, in Serbia, eggs are eaten on a daily basis, so attention must be focused on optimizing the impact caused by the process of retail and household consumption [39].

**Figure 2:**
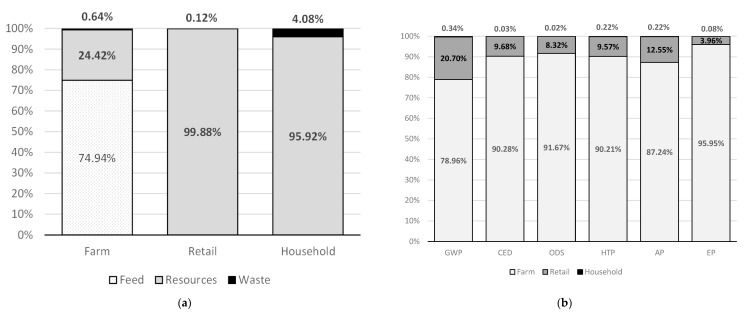
Life cycle of the egg supply chain; (**a**) Relative contributions (in %) to global warming potential from processes involved in the three subsystems—farms, retailers and households; (**b**) Relative contributions (in %) to environmental impacts from the three subsystems—farms, retailers and households. Legend: Resources—water and all types of energy; Waste—all types of waste and wastewater; GWP—global warming potential; CED—cumulative energy demand; ODS—ozone depletion substances; HTP—human toxicity potential, AP—acidification potential, EP—eutrophication potential.

The results show that there are statistically significantly different influences by subsystems. Each of the examined subsystems in the egg supply chain has its role in the impact on GWP, and which can be seen through the production and consumption of animal feed, consumption of natural resources and generation of all types of waste and wastewater (Figure 2a). Animal feed was the most influential (74.94%) in the production on farm subsystem, which is in line with the results of other LCA studies that analyzed the contributions of all processes in different production systems. Consumption of natural resources had the greatest impact on GWP in the retail (99.88%) and household consumption (95.92%) subsystems. In addition to the above, the consumption of natural resources in the form of electricity and fossil fuels for transport had a significant impact on the farm subsystem (24.42%). Household waste had a greater impact on GWP (4.08%) compared to the retail subsystem (0.12%) and the farm (0.64%), which is logical given that household egg waste in Serbia is not recycled.

Activities on the farm contributed the most to all examined environmental impacts individually (78.96%) GWP, (90.28%) CED, (91.67%) ODS, (90.21%) HTP, (87.24%) AP, (95.95%) EP (Figure 2b). This was followed by retail (20.70%) GWP, (9.68%) CED, (8.32%) ODS, (9.57%) HTP, (12.55%) AP, (3.96%) EP and households with the lowest contribution (0.34%) GWP, (0.03%) CED, (0.02%) ODS, (0.22%) HTP, (0.22%) AP, (0.08%) EP.

### 3.2. Assessment of Environmental Aspects in the Comparison of Customer–Supplier Interaction

**Figure 3:**
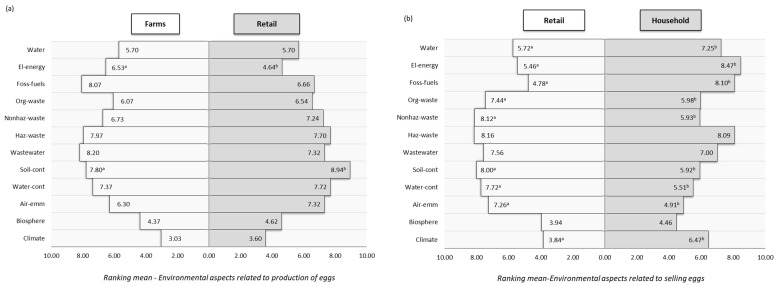
Environmental aspects related to the egg chain; (**a**) Interaction between farmers and retailers; (**b**) Interaction between retailers and households’ consumers. Note that different letters show statistically significant difference *p* < 0.05. Legend: El-energy—electric energy, Foss-fuel—fossil fuel, Org-waste—organic waste, Nonhaz-waste—nonhazardous waste, Haz-waste—hazardous waste, Soil-cont—soil contamination, Water-cont—water contamination, Air-emm—air emission.

Out of the 12 identified environmental aspects related to egg production, wastewater discharge (8.20), fossil fuel use (8.07) and hazardous waste management (7.97) were identified as dominant from the farm point of view (Figure 3a). The process of egg production begins with adequate preparation of the facility for the introduction of laying hens, which includes thorough cleaning, washing and disinfection. The producers considered the most significant environmental impacts arising from these activities to be the consumption of water as a natural resource, the use of cleaning agents, the generation of hazardous packaging and the discharge of wastewater after sanitation. In addition to the above, wastewater can be generated from the production plant during feeding and watering of laying hens, after washing eggs and from the area for storage of waste material and manure. Effluent formation due to poor waste and manure management has a negative environmental impact on surface- and groundwater due to high organic load [40]. All activities on the farm generate large amounts of organic and inorganic waste. Inadequate disposal of various types of packaging, cleaning agents, veterinary preparations and agrochemicals has a significant environmental impact on soil and water contamination. Additionally, farmers considered the activity of fossil fuel consumption for transport, the intensity of which depended on the total GHG emissions, as significant in all described phases of the producer subsystem. Retailers shared a similar opinion regarding water pollution (7.72) and the generation of hazardous waste (7.70), but singled out land contamination as having the most significant environmental impact (8.94). Significant statistical differences (*p* < 0.05) were found for two aspects of the environment impact related to egg production evaluated by producers and retailers.

Of the 12 identified environmental aspects related to egg retail (Figure 3b), retailers singled out the management of hazardous (8.16) and non-hazardous (8.12) waste and soil contamination (8.00) as the most significant. Consumers shared the opinion of retailers regarding hazardous waste (8.09) but also pointed out the use of fossil fuels for transport (8.10) and electricity consumption (8.47) as having very important environmental impacts. Retail plays a very important role in the handling and storage of eggs, and activities during transport, preservation of the cold chain and waste management have specific environmental impacts [41]. The impact of the retail sector on total GHG emissions is somewhat limited, which is confirmed by our LCA results (Figure 2). In retail facilities, electricity is mainly used for the operation of refrigerated display cases and their lighting, while leakage of refrigerants from these devices is the most important direct source of emissions [42]. The Serbian current ordinance on egg quality is in line with European regulations, but does not define the temperature interval at which eggs should be stored until the moment of purchase by consumers. It is up to producers and retail chains to assess the level of risk related to storage conditions and select an appropriate regime. Some retail chains in Serbia have started adopting recommendations for storing eggs at ambient temperature, while others are advocating that eggs remain in the cold chain [43].

Another important source of emissions in the retail sector was the generation of waste, recognized by both retailers and consumers. Organic waste means the annual quantity of broken eggs and the annual quantity of eggs that are not sold within the prescribed period of use. Inorganic waste means all materials used for packaging, transport and storage [41]. The transport of eggs from producers to retail outlets was of great importance for the total GHG emissions, especially over long distances. Important aspects of this activity were the mode of transport and the type of vehicle. These can differ significantly in intensity and, thus, in emissions, which consumers recognize as a significant environmental aspect. Transporting eggs from producers to retail facilities or distribution centers is an inefficient process due to slow vehicle speeds, perhaps with many stops, and low vehicle load factors [42]. The environmental impacts of nine activities in the egg supply chain at the retail level were assessed differently by retailers and household consumers with significant statistical differences (*p* < 0.05).

### 3.3. Application of the QFDE Method in the Egg Supply Chain

**Figure 4:**
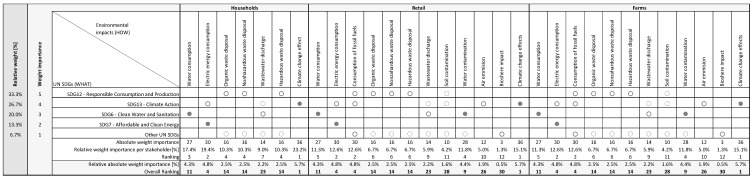
Environmental houses of quality (HOQ) in the egg chain; Legend: UN—United Nations; SDG—Sustainable Development Goals; ● ‘strong relationship’ = 9, ○ ‘moderate’ = 3, ◌ ‘weak relationship’ = 1 and blank = ‘non-existent’ or ‘zero’.

The UN SDGs highlighted by the chain actors as being the most important to achieve were responsible household consumption and responsible egg production on the farm (33.3%), followed by mitigation of the impact of all activities throughout the chain on climate change (26.7%), followed by more efficient use of water (20.0%) and energy (13.3%) (Figure 4).

The first observed subsystem, household consumption, was related to the recognition of environmental impacts by consumers during the storage and preparation of eggs, which are correlated with the achievement of ranked UN SDGs. As the most important, household consumers singled out the effects on climate change (23.2%) and electricity consumption (19.4%).

The observed relationships between the established UN SDGs and activities in the retail and production on farms gave the same results. Retailers and egg producers believed that the most important activities that impacted the ranked UN SDGs were the effect on climate change (15.1%), of fossil fuel consumption (12.6%) and electricity consumption (12.6%) that arose from their activities.

The observed relative absolute importance of weight throughout the egg supply chain showed that the most important environmental impacts were the effect on climate change (5.7%), electricity consumption (4.8%) and fossil fuel consumption (4.8%).

### 3.4. Implications in Practice

Our analysis of current practice and application of a two-way model to observe the egg chain confirmed differences in perceptions towards the environment among producers, retailers and household consumers. All actors along the entire egg chain should engage and communicate properly to identify and reduce environment hazards/risks caused by the egg chain. In addition to the critical points, this research identified three opportunities for mitigating the egg chain’s environmental impacts: optimization of feed ingredients; modification of the use of electricity and fossil fuels for egg transport; and recycling household egg waste at consumer level.

## 4. Conclusions

The research provides an additional approach to assessing environmental performance (GWP, CED, ODS, HTP, AP and EP) in the egg chain, including chain actors in three subsystems (producers, retailers and household consumers). The LCA of each individual subsystem was performed and for the first time the environmental impact of the whole egg supply chain was presented. The results of the research confirm the first working hypothesis that there are differences in environmental impact between the actors in the chain. The entire supply chain of table eggs emits 3.33 kg CO_2_eq/kg egg, 29.01 MJ_e_/kg, 0.17 mg R11_e_/kg, 1.15 kg 1.4 DB_e_/kg, 17.76 g SO2_e_/kg and 27, 79 g PO4_e_/kg. Egg producer activities at farm level had the greatest environmental impact through the provision of feed for laying hens (74.94%) and the use of natural resources (24.42%), and made the greatest contribution to the impact of each individual environmental indicator.

By analyzing the perceptions of the environmental impacts of all actors, we concluded that actors differently perceive the environmental impacts along the entire supply chain of eggs, which confirms the second working hypothesis. Comparison of environmental impacts viewed as important by actors from farm and retail showed that farmers thought the use of fossil fuels, wastewater management and hazardous waste handling were important. In retail, they shared the opinion of farmers regarding the generation of hazardous waste, but they pointed to the pollution of land and water as very important aspects. The retail-household interaction showed that retailers thought the management of non-hazardous waste and soil contamination were important, while household consumers thought the use of electricity and fossil fuels had high environmental impacts. The management of hazardous waste was equally important for both these actors in the egg chain. The farm subsystem did not recognize the negative environmental impact caused by provision of food for laying hens, while retail did not rank the consumption of electricity and fossil fuels for transport as the most important. Household consumers did not recognize the importance of two types of waste generated during the preparation of eggs for consumption. The results obtained indicate the need to launch environmental initiatives aimed at education and awareness raising through all subsystems individually, in order to identify the most important environmental impacts and take measures to reduce them. Finally, the application of the results of the QFDE method can improve the environmental performance of each observed subsystem individually, but can also take into account the environmental perceptions of all stakeholders throughout the chain in achieving the goals towards a sustainable egg supply chain.

Limitations of this study: Retailers did not report on refilling or replacing refrigerants. Only five households reported refilling refrigerants in the last 12 months, so this was not included in the study.

## Figures and Tables

**Table 2:** Summary of inventory data sources considered in this study.

		GWP (kgCO_2_/IU)	CED (MJ_e_/IU)	ODS (kg R11_e_/IU)	HTP (kg 1.4 DB_e_/IU)	AP (kg SO2_e_/IU)	EP (kg PO4_e_/IU)	Source
Energy	Electricity (Serbian profile)	1.099	8.31 × 10^−1^	1.78 × 10^−9^	2.28 × 10^−1^	3.42 × 10^−3^	1.96 × 10^−3^	Serbian legislation [32]
	Liquefied petroleum gas	2.961	1.56	1.97 × 10^−7^	2.53 × 10^−1^	3.39 × 10^−3^	6.51 × 10^−4^	CCaLC database [31]; Serbian legislation [32]
	Diesel	2.76	4.01 × 10^−1^	3.31 × 10^−7^	3.87 × 10^−1^	6.10 × 10^−3^	8.82 × 10^−3^	CCaLC database [31]; Serbian legislation [32]
	Petrol	2.209	5.68 × 10^−1^	4.71 × 10^−7^	4.24 × 10^−1^	7.96 × 10^−3^	9.37 × 10^−3^	CCaLC database [31]; Serbian legislation [32]
	Natural gas	1.852	4.29 × 10^−1^	1.45 × 10^−9^	8.33 × 10^−1^	2.54 × 10^−2^	1.47 × 10^−3^	CCaLC database [31]; Serbian legislation [32]
	Wood (chips and logs)	18	35	1.21 × 10^−6^	24.3	1.44 × 10^−1^	7.10 × 10^−2^	CCaLC database [31]; Serbian legislation [32]
	Pellets	0.131	1.49	7.05 × 10^−9^	253	7.50 × 10^−2^	1.45 × 10^−3^	CCaLC database [31]; Serbian legislation [32]
Feed	Maize for feed	4.90 × 10^−1^	2.02	3.50 × 10^−8^	1.27 × 10^−1^	3.08 × 10^−3^	4.21 × 10^−3^	CCaLC database [31]
	Wheat for feed	6.40 × 10^−1^	2.02 × 10^−1^	4.13 × 10^−8^	4.22 × 10^−1^	4.32 × 10^−3^	7.55 × 10^−3^	CCaLC database [31]
	Soybean for feed	9.00 × 10^−1^	2.59	4.33 × 10^−8^	3.55 × 10^−1^	6.65 × 10^−3^	1.50 × 10^−2^	CCaLC database [31]
	Sunflower for feed	1.02	35.9	7.63 × 10^−8^	4.05 × 10^−1^	4.64 × 10^−3^	1.31 × 10^−2^	CCaLC database [31]
	Barley for feed	4.85 × 10^−1^	1.96	3.19 × 10^−8^	3.25 × 10^−1^	3.83 × 10^−3^	8.57 × 10^−3^	CCaLC database [31]
	Feed mixes for chicken	4.58 × 10^−1^	12.5	4.55 × 10^−8^	3.27 × 10^−1^	4.50 × 10^−3^	9.68 × 10^−3^	CCaLC database [31]
Packaging materials	Carton	8.62 × 10^−1^	21.9	9.57 × 10^−8^	1.72 × 10^−4^	2.86 × 10^−3^	2.15 × 10^−3^	CCaLC database [31]
	Styrofoam	3.30	0.00	0.00	1.50 × 10^−3^	1.08 × 10^−2^	9.30 × 10^−4^	CCaLC database [31]
	Polyvinyl chloride (PVC)	3.22	0.00	0.00	1.15 × 10^−3^	1.39 × 10^−2^	1.19 × 10^−3^	CCaLC database [31]
	Polyethylene (HDPE)	3.26	0.00	0.00	1.54 × 10^−3^	1.52 × 10^−2^	1.18 × 10^−3^	CCaLC database [31]
Cleaning agents	Acid chemicals	1.1	22.8	6.69 × 10^−8^	8.91 × 10^−1^	5.27 × 10^−3^	3.70 × 10^−3^	CCaLC database [31]
	Alkaline chemicals	3.17	13.4	1.04 × 10^−7^	2.10 × 10^−1^	1.59 × 10^−2^	3.35 × 10^−4^	CCaLC database [31]
Water	Tap water at user	0.0005	0.00	6.76 × 10^−12^	2.44 × 10^−5^	1.40 × 10^−6^	4.45 × 10^−7^	CCaLC database [31]
	Well water	0.0003	6.17 × 10^−3^	1.61 × 10^−11^	0.0001847	1.39 × 10^−6^	8.73 × 10^−7^	CCaLC database [31]
Waste	Manure	0.004	5.30 × 10^−2^	4.02 × 10^−10^	1.13 × 10^−1^	5.68 × 10^−4^	1.12 × 10^−3^	CCaLC database [31]
	Waste-wood	1.42	0.00	2.78 × 10^−9^	2.83 × 10^−3^	5.35 × 10^−4^	2.02 × 10^−4^	CCaLC database [31]
	Waste-plastic	0.071	0.00	2.782 × 10^−9^	2.30 × 10^−3^	2.44 × 10^−4^	1.06 × 10^−4^	CCaLC database [31]
	Waste-paper	0.008	0.00	4.1 × 10^−12^	1.68 × 10^−3^	1.94 × 10^−6^	2.03 × 10^−7^	CCaLC database [31]
	Waste-carton	0.119	0.00	2.128 × 10^−9^	1.55 × 10^−6^	4.22 × 10^−5^	1.03 × 10^−5^	CCaLC database [31]
	Waste-Styrofoam	0.008	0.00	4.1 × 10^−12^	1.68 × 10^−3^	1.94 × 10^−6^	2.03 × 10^−7^	CCaLC database [31]
	Food waste (eggshells)	0.513	3.79 × 10^−1^	2.78 × 10^−9^	2.41 × 10^−3^	3.56 × 10^−4^	1.28 × 10^−3^	CCaLC database [31]
	Food waste (oil)	0.513	3.79 × 10^−1^	2.78 × 10^−9^	2.41 × 10^−3^	3.56 × 10^−4^	1.28 × 10^−3^	CCaLC database [31]
	Landfill-municipal waste	0.513	3.79 × 10^−1^	2.78 × 10^−9^	2.41 × 10^−3^	3.56 × 10^−4^	1.28 × 10^−3^	CCaLC database [31]
	Wastewater-industrial treatment	0.00241	6.49 × 10^−3^	2.20 × 10^−10^	1.20 × 10^−3^	6.92 × 10^−5^	2.60 × 10^−5^	CCaLC database [31]

GWP—Global warming potential; CED—Cumulative energy demand; ODS—ozone depletion substances; HTP—Human toxicity potential; AP—Acidification potential; EP—Eutrophication potential. IU—Inventory unit (may be kg or L or kWh).

**Table 3:** Environmental impact assessment results associated with the production of 1 kg of eggs.

Impact Category	Unit	Farm	Retail	Household	Supply Chain
GWP	(kgCO_2_eq/FU)	2.63 ± 0.80 ^a^	0.69 ± 0.30 ^b^	0.0113 ± 0.0071 ^c^	3.33 ± 1.11
CED	(MJ_e_/FU)	26.20 ± 8.78 ^a^	2.81 ± 0.88 ^b^	0.0096 ± 0.0051 ^c^	29.01 ± 9.67
ODS	(mg R11_e_/FU)	0.15 ± 0.05 ^a^	0.01 ± 0.01 ^b^	0.00003 ± 0.00001 ^c^	0.17 ± 0.05
HTP	(kg 1.4 DB_e_/FU)	1.04 ± 0.28 ^a^	0.11 ± 0.05 ^b^	0.0025 ± 0.0012 ^c^	1.15 ± 0.34
AP	(g SO2_e_/FU)	15.49 ± 4.36 ^a^	2.23 ± 1.00 ^b^	0.0382 ± 0.0114 ^c^	17.76 ± 5.4
EP	(g PO4_e_/FU)	26.67 ± 7.70 ^a^	1.10 ± 0.48 ^b^	0.0229 ± 0.0118 ^c^	27.79 ± 8.21

GWP—Global warming potential; CED—Cumulative energy demand; ODS—ozone depletion substances; HTP—Human toxicity potential; AP—Acidification potential; EP—Eutrophication potential; FU—Functional unit (1 kg of eggs). Statistically significant difference presented in different letters (^a,b,c^) (*p* < 0.05).

## Data Availability

The data presented in this study are available on reasonable request from the corresponding author.
